# Predictive and Prognostic Value of Selected MicroRNAs in Luminal Breast Cancer

**DOI:** 10.3389/fgene.2019.00815

**Published:** 2019-09-11

**Authors:** Maria Amorim, João Lobo, Mário Fontes-Sousa, Helena Estevão-Pereira, Sofia Salta, Paula Lopes, Nuno Coimbra, Luís Antunes, Susana Palma de Sousa, Rui Henrique, Carmen Jerónimo

**Affiliations:** ^1^Cancer Biology and Epigenetics Group, IPO Porto Research Center (CI-IPOP), Portuguese Oncology Institute of Porto (IPO Porto), Porto, Portugal; ^2^Master in Oncology, Institute of Biomedical Sciences Abel Salazar–University of Porto (ICBAS-UP), Porto, Portugal; ^3^Department of Pathology, Portuguese Oncology Institute of Porto, Porto, Portugal; ^4^Department of Pathology and Molecular Immunology, Institute of Biomedical Sciences Abel Salazar–University of Porto (ICBAS-UP), Porto, Portugal; ^5^Department of Medical Oncology, Portuguese Oncology Institute of Porto, Porto, Portugal; ^6^Department of Epidemiology, Portuguese Oncology Institute of Porto, Porto, Portugal

**Keywords:** Breast cancer, luminal subtype, endocrine therapy, endocrine resistance, biomarkers, microRNAs

## Abstract

Breast cancer (BrC) is the most frequent malignancy and the leading cause of cancer death among women worldwide. Approximately 70% of BrC are classified as luminal-like subtype, expressing the estrogen receptor. One of the most common and effective adjuvant therapies for this BrC subtype is endocrine therapy. However, its effectiveness is limited, with relapse occurring in up to 40% of patients. Because microRNAs have been associated with several mechanisms underlying endocrine resistance and sensitivity, they may serve as predictive and/or prognostic biomarkers in this setting. Hence, the main goal of this study was to investigate whether miRNAs deregulated in endocrine-resistant BrC may be clinically relevant as prognostic and predictive biomarkers in patients treated with adjuvant endocrine therapy. A global expression assay allowed for the identification of microRNAs differentially expressed between luminal BrC patients with or without recurrence after endocrine adjuvant therapy. Then, six microRNAs were chosen for validation using quantitative reverse transcription polymerase chain reaction in a larger set of tissue samples. Thus, *miR-30c-5p*, *miR-30b-5p*, *miR-182-5p*, and *miR-200b-3p* were found to be independent predictors of clinical benefit from endocrine therapy. Moreover, *miR-182-5p* and *miR-200b-3p* displayed independent prognostic value for disease recurrence in luminal BrC patients after endocrine therapy. Our results indicate that selected miRNAs’ panels may constitute clinically useful ancillary tools for management of luminal BrC patients. Nevertheless, additional validation, ideally in a multicentric setting, is required to confirm our findings.

## Introduction

Breast cancer (BrC) is the second most common cancer worldwide and the most frequent cancer among women. Despite advances in screening, early diagnosis, and treatment strategies, BrC still constitutes the leading cause of cancer-related death among women (Bray et al., 2018). BrC is a highly heterogeneous disease with distinct biological features and clinical outcomes. Based on gene expression profiling, BrC is often classified into four well-established intrinsic subtypes (**Table 1**) (Sørlie, 2004; Parker et al., 2009). However, due to logistic and economical constraints, surrogate approaches have been developed for routine clinical practice, using widely available immunohistochemistry (IHC) assays for estrogen receptor (ER), progesterone receptor (PR), and Ki-67 *index*, together with IHC and/or *in situ* hybridization for human epidermal growth factor 2 receptor (HER2) overexpression/amplification (Senkus et al., 2015).

**Table 1:** Breast cancer molecular subtypes characterization (Perou et al., 2000; Sørlie et al., 2001; Oh et al., 2006; Eroles et al., 2012; Haque et al., 2012; Network, 2012; Howell, 2013; Zhang et al., 2014a; Senkus et al., 2015).

Breast cancer subtypes		Clinicopathological surrogate markers	Signature genes	Adjuvant systemic therapeutic options
**Luminal A**		ER^+^ PR high^1^ HER2^-^ Ki-67 low^2^	*ESR1* and/or *PGR*, *KRT8*/18, *GATA3*, *XBP1*, *FOXA1*, and *ADH1B*	ET alone in most of cases + ChT if high tumor burden (≥N3, ≥T3)
**Luminal B**	**HER2^-^**	ER^+^ HER2^-^ Ki-67 high or PR low	*ESR1* and/or *PGR*, *KRT*8/18, *FGFR1*, *ERBB1*, *MKI67* and/or *CCNE1*, *CCNB1*, and *MYBL2*	ET + ChT for the most of cases
	**HER2^+^**	ER^+^ HER2^+^ Any Ki-67 Any PR		ChT + anti-HER2 + ET for all patients
**Basal-like**		ER^-^ PR^-^ HER2^-^	*KRT*5/6, *KRT*17, *ERBB1* and/or *KIT*, FOXC1, TP63, *CDH3*, VIM, and *LAM*	ChT
**HER2-enriched**		HER2^+^ ER^-^ PR^-^	*ERBB2* and *GRB7*	ChT + anti-HER2

^1^Suggested cutoff value is 20%. ^2^Ki-67 scores should be interpreted in the light of local laboratory median values. ER, estrogen receptor; PR, progesterone receptor; HER2, human epidermal growth factor receptor 2; ESR1, estrogen receptor 1; PGR, progesterone receptor; KRT, keratin; GATA3, GATA binding protein 3; XBP1, X-box binding protein 1; FOX, forkhead box; ADH1B, alcohol dehydrogenase 1B (Class I), beta polypeptide; FGFR1, fibroblast growth factor receptor 1; ERBB, Erb-B2 receptor tyrosine kinase; MKI67, marker of proliferation Ki-67; CCN, cyclin; MYBL2, MYB proto-oncogene like 2; MYBL2, MYB proto-oncogene like 2; KIT, KIT proto-oncogene receptor tyrosine kinase; TP63, tumor protein P63; CDH, cadherin; VIM, vimentin; LAM, laminin; GRB7, growth factor receptor bound protein 7; ChT, chemotherapy; ET, endocrine therapy; N, nodal stage; T, tumor size.

In addition to surgery, therapeutic strategies for BrC patients include neoadjuvant, adjuvant, and palliative treatments. Adjuvant systemic therapy, aiming to prevent BrC recurrence by eradicating micrometastases present at diagnosis, includes three modalities: chemotherapy, anti-HER2 therapy (e.g., trastuzumab), and endocrine therapy (ET). ER and HER2 *status* are used as predictive factors to select patients for specific adjuvant therapies (**Table 1**). ET, which blocks ER activation, is recommended for patients with ER-positive disease, to stop or slow the growth of hormone-sensitive BrC (Curigliano et al., 2017). Most luminal A BrC tumors do not require adjuvant chemotherapy, except those with the highest risk of relapse, whereas most luminal B tumors, especially those with HER2 overexpression, benefit from chemotherapy in addition to trastuzumab (Slamon et al., 2011). Although ET results in substantial improvement of patients’ outcome, resistance to treatment is a major hurdle (Zhang et al., 2014a), affecting 30–40% of ER-positive BrC patients, with all those treated in the metastatic setting eventually progressing (Normanno et al., 2005; Murphy and Dickler, 2016). According to the 3rd ESO–ESMO International Consensus Guidelines, endocrine resistance may be defined as primary endocrine resistance, when patients relapse within the first 2 years of adjuvant ET, or as secondary (acquired) endocrine resistance, when patients relapse while on adjuvant ET after the first 2 years of treatment or within 12 months after completing treatment (Cardoso et al., 2017).

MicroRNAs (miRNAs), a class of small (∼22 nucleotides) non-coding single-stranded RNAs, have shown promise for assisting in clinical management of BrC as diagnostic, prognostic, or predictive biomarkers (Amorim et al., 2016), namely, through assessment in liquid biopsies (plasma, serum, and urine) (Schwarzenbach et al., 2014). Indeed, several studies have associated miRNAs deregulation with endocrine resistance and prognosis in luminal BrC (Rodriguez-Gonzalez et al., 2011; Muluhngwi and Klinge, 2015; Barbano et al., 2017; Muluhngwi and Klinge, 2017). Whereas decreased ER expression and endocrine resistance may be due to *miR-221/222* overexpression (Zhao et al., 2008; Rao et al., 2011; Wei et al., 2014; Song et al., 2017), *miR-342-3p* expression positively correlated with ER mRNA transcript levels, being downregulated in tamoxifen-refractory BrC (Cittelly et al., 2010). Moreover, miRNAs regulating growth, survival, and apoptosis of BrC cells may also be implicated in loss of responsiveness to ET by endowing tumor cells with alternative proliferative and survival stimuli (Thiantanawat et al., 2003). Indeed, *miR-519a* associated with worse prognosis in luminal BrC patients, directly targeting the transcripts of *cyclin dependent kinase inhibitor 1A* (*CDKN1A*) and *phosphatase and tensin homolog* (*PTEN*), allowing for enhanced signaling of the *phosphoinositide3-kinase* (PI3K) growth and survival pathway (Ward et al., 2014) and reducing sensitivity and tumor cell apoptosis in response to apoptotic stimuli (Breunig et al., 2017). Furthermore, miRNA-mediated endocrine resistance might be related with epithelial-to-mesenchymal transition (EMT) and metastatic potential of BrC cells, as members of the *miR-200family(miR-200f)*, which act as major regulators of EMT, were found downregulated in endocrine-resistant BrC *vs*. endocrine-sensitive cell lines (Burk et al., 2008; Manavalan et al., 2013).

Herein, we aimed to identify miRNAs that might predict endocrine resistance in luminal BrC patients undergoing ET, by comparing expression levels between BrC samples of patients that developed endocrine resistance with those that did not, after long-term follow-up. Expression levels of the miRNAs identified might allow for stratification of luminal BrC cases into a low-risk patient subgroup, for which additional adjuvant systemic treatment can be safely omitted, and a high-risk group comprising patients at high risk for recurrence, allowing for detection of resistance to ET at an early stage.

## Materials and Methods

### Patients and Samples Collection

For this study, 139 BrC tissue samples were prospectively collected, after informed consent, from patients with luminal BrC and without metastasis at diagnosis, aged between 41 and 75 years, submitted to adjuvant ET (with or without other adjuvant modalities), after first-line surgical treatment, from 1995 to 2002 at the Portuguese Oncology Institute of Porto (IPO-Porto). Furthermore, 26 normal breast tissue samples were collected from reduction mammoplasties of contralateral breast from BrC patients. All these specimens were obtained from patients without BrC hereditary syndrome and no evidence of preneoplastic/neoplastic lesions. After surgical resection, samples were immediately frozen at −80°C. Relevant clinical and pathological data were retrieved from patients’ charts. Five-micrometer frozen sections were cut and stained with hematoxylin–eosin (H&E) staining for confirmation of BrC by an experienced pathologist, ensuring that samples contained at least 70% of tumor cells, and confirm that tissues obtained from reduction mammoplasties harbored normal epithelial cells. This study was approved by institutional ethical committee (CES-IPOFG-120/015).

### BrC Subtyping

IHC was performed to identify the molecular subtype of each tumor tissue included in this study. Commercially available antibodies were used for ER (Clone 6F11, mouse, Leica), PR (Clone 16, mouse, Leica), HER2 (Clone 4B5, rabbit, Roche), and Ki-67 (Clone MIB-1, mouse, Dako). IHC was carried out in BenchMark ULTRA (Ventana, Roche) using ultraView Universal DAB Detection Kit (Ventana, Roche) according to the manufacturer’s instructions. Each case was evaluated by an experienced pathologist; it was classified according to the College of American Pathologists recommendations (Fitzgibbons et al., 2014) and categorized according to ESMO guidelines (Senkus et al., 2015). Cutoffs for Ki-67 and PR expression were set at 15% and 25% of positive cells, respectively, according to the optimized protocols of Department of Pathology.

### RNA Extraction From Fresh Frozen Tissues

Total RNA was extracted from fresh frozen tissues using the TRIzol^®^ Reagent (Invitrogen, Carlsbad, CA, USA) according to the manufacturer’s recommendations. RNA concentrations and purity ratios were ascertained using a NanoDrop Lite spectrophotometer (NanoDrop Technologies, Wilmington, DE, USA), and RNA samples were stored at −80ºC.

### MiRNA cDNA Synthesis

cDNA synthesis was performed in a Veriti^®^ Thermal Cycler (Applied Biosystems, Foster City, CA, USA) using miRCURY LNA™ Universal RT microRNA PCR (Exiqon, Vedbaek, Denmark) following the manufacturer’s instructions. cDNA samples were then stored at −20ºC.

### Global Focus MiRNA PCR Panel

Global miRNAs’ expression was evaluated using a Cancer Focus microRNA PCR Panel, 384-well (V4.R) (Exiqon). Each plate, besides containing 80 lyophilized LNA™ miRNA primer sets focusing on cancer-relevant human miRNAs, also contained interplate calibrators, candidate reference genes [miRNAs and small nuclear RNAs (snRNAs)], and one water blank. In each well, 0.05 μl of cDNA previously synthesized, 5 μl of SYBR^®^ Green master mix (Exiqon), and 4.95 μl of nuclease-free water (Exiqon) were added. Quantitative reverse transcription polymerase chain reactions (RT-qPCR) were performed in the LightCycler 480 instrument (Roche Diagnostics, Manheim, Germany) according to the following conditions: 95ºC for 10 min and 45 cycles at 95ºC for 10 s and 60ºC for 1 min.

The median values of *miR-103a-3p*, *miR-107*, *miR-191-5p*, and *SNORD38B* were used for normalization, as these genes were the most stably expressed candidate reference genes (**Supplementary Figure 1**). Differences in expression values for target miRNAs were calculated using the 2^−ΔΔCT^ method. The selection of deregulated miRNAs for further validation was performed considering prominent fold change, good sensitivity for qRT-PCR detection (Ct values, in general, below 30), and novelty.

### Individual Assays

Initially, cDNA samples were diluted 80× in sterile distilled water (B. Braun, Melsungen, Germany). Then, on ice, *per* each well of a 384-well plate, the following were added: 5 μl of NZYSpeedy qPCR Green Master Mix (2×) (NZYTECH, Portugal), 1 μl of miRNA specific primer mix (microRNA LNA™ PCR primer set, Exiqon), and 4 μl of previously diluted cDNA. Each amplification reaction was performed in triplicate on a LightCycler 480 instrument (Roche Diagnostics, Manheim, Germany). Each plate also contained two negative template controls. RT-qPCR protocol consisted of a denaturation step at 95ºC for 2 min, followed by 40 amplification cycles at 95ºC for 5 s and 60ºC for 20 s. Melting curve analysis was performed according to the instrument’s manufacturer’s recommendations.

*SNORD38B* was used as a reference gene for data normalization, as this gene was the most stably expressed over the whole range of the samples used for the global expression assay. Notwithstanding, the stability of *SNORD38B* expression was empirically validated in additional samples. Relative miRNA expression in each sample was calculated by the 2^−ΔΔCT^ method (the target sequences of mature miRNAs analyzed are provided in **Supplementary Table 1**).

### Statistical Analysis

Statistical analysis was performed using SPSS software (SPSS Version 24.0, Chicago, IL), and two-tailed *p* values were considered statistically significant when *p* < 0.05. Graphs were constructed using GraphPad 6 Prism (GraphPad Software, USA).

#### MiRNA Expression Analysis

Fold changes for single miRNAs were calculated using the 2^−ΔΔCT^ method (Livak and Schmittgen, 2001).

#### Association Between MiRNA Expression and Clinicopathological Features

To ascertain statistical significance for continuous variables, comparisons were made between independent samples and non-parametric Mann–Whitney *U* tests were performed. Spearman nonparametric correlation test was performed to assess the association between continuous variables. Chi-square test or Fisher’s exact test were used as appropriate to compare proportions between two groups.

#### Survival Analysis

Some clinicopathological features were grouped, including pT stage (T1 and T2 and T3 and T4), pN stage (N0 and N1 and N2 and N3), and grade [grade (G)1 and G2 and G3] (Lakhani, 2012). Age was categorized into four groups (≤44, 45–64, 65–74, and ≥75), and miRNA expression levels were categorized according to 25th or 75th percentile. All survival analyses were restricted to 15 years of follow-up. Cox regression univariable and multivariable models were computed to assess standard clinicopathological variables and miRNA prognostic value. Hazard ratios (HRs) along with respective 95% confidence interval (95% CIs) were reported. Multivariable Cox models only included the statistically significant variables. Kaplan–Meier with log rank test was used to construct and compare survival curves according to categorized miRNA expression levels. Endocrine resistance-free survival (ERFS) was defined as the time between surgery and the recurrence dates. Recurrences occurring after 12 months of completing ET were not considered events for this analysis. Disease-free survival (DFS) was defined as the time between surgery date and recurrence date. Distant metastasis-free survival (DMFS) was defined as the time between surgery and the development of distant metastases. For prognostic assessment of miRNAs combined in panels, the miRNAs that remained statistically significant in multivariable analysis were differently combined, considering the same categories used in previous survival analysis (expression above or below P25). The best panels were selected based on the individual markers value in the Cox model: better HR, smaller 95% CI and *p* value, as well as value in stratified analysis.

## Results

### Characteristics of Study Populations

The discovery cohort (*n* = 16), used for global expression assay analysis, consisted of four luminal A and four luminal B tumors from BrC patients who relapsed, and the same number of patients who did not relapse after adjuvant ET. Patients who relapsed during adjuvant ET or within the first 12 months of completing adjuvant ET were considered endocrine-resistant (**Table 2**).

**Table 2:** Clinical and pathological data of luminal tumors included in the discovery cohort.

	Molecular subtype	Age at diagnosis	Grade	Stage	ChT	RT	Recurrency site	Endocrine-resistant
**Patients who relapsed**	**Luminal A**	82	G2	IIIA	NO	NO	Liver	YES
		41	G3	IIA	YES	YES	Bone	YES
		60	UNKN	IA	NO	YES	Contralateral breast	NO
		43	G2	IIB	YES	YES	Lymph nodes	NO
	**Luminal B**	65	G3	IIIC	YES	YES	Lung	YES
		63	G2	IIIA	NO	YES	Bone	YES
		67	G2	IIB	NO	NO	Bone	NO
		66	G3	IIIA	NO	NO	Locoregional	NO
**Patients who did not relapse**	**Luminal A**	70	G3	IIB	NO	YES	n.a.	n.a.
		68	G2	IIB	NO	YES		
		69	G2	IIIA	NO	NO		
		69	G2	IA	NO	YES		
	**Luminal B**	65	G3	IIIC	YES	YES		
		72	G3	IIIC	NO	YES		
		70	G1	IIB	NO	YES		
		73	G1	IIIC	NO	YES		

ChT, chemotherapy; RT, radiotherapy; UNKN, unknown; n.a., not applicable.

The validation cohort was composed of a total of 149 subjects, comprising 123 luminal BrC and 26 normal breast tissues. Among 34 cancer patients that recurred during follow-up time, 20 were considered endocrine-resistant. Clinical and pathological characteristics of patients and controls included in this study are shown in **Table 3**. Endocrine-sensitive and endocrine-resistant groups did not significantly differ concerning age distribution (*p* = 0.136). As expected, most of the endocrine-resistant cases were classified as luminal B (*p* = 0.011) and depicted high Ki-67 *index* (*p* = 0.001). Moreover, this group also showed a higher number of high-grade (G3) cases (*p* = 0.027). For the remaining clinicopathological features or treatment modalities, no significant differences were depicted.

**Table 3:** Clinical and pathological data of luminal tumors and normal breast samples included in the validation cohort.

Clinipathological features	Endocrine-sensitive	Endocrine-resistant	NBr
**Patients** (n)	103	20	26
**Age median** (range)	61 (43–73)	59 (41–75)	54 (40–70)
	61 (41–75)		
**Molecular subtype (%)** Luminal A Luminal B	47 (45.6) 56 (54.4)	3 (15.0) 17 (85.0)	n.a.
**Histological type (%)** Invasive carcinoma of NST (IDC) Invasive lobular carcinoma Other special subtype carcinoma Mixed type carcinoma	89 (86.4) 5 (4.8) 1 (1.0) 8 (7.8)	17 (85.0) 2 (10.0) 1 (5.0) 0 (0.0)	n.a.
**Progesterone receptor status (%)** Positive Negative	85 (82.5) 18 (17.5)	13 (65.0) 7 (35.0)	n.a.
**HER2 receptor status (%)** Positive Negative	9 (8.7) 94 (91.3)	5 (25.0) 15 (75.0)	n.a.
**Ki-67 index (%)** <15% >15% UNKN	78 (75.7) 20 (19.4) 5 (4.9)	6 (30.0) 10 (50.0) 4 (20.0)	n.a.
**Grade (%)** G1 G2 G3 Not determined	16 (15.5) 53 (51.5) 28 (27.2) 6 (5.8)	0 (0.0) 8 (40.0) 10 (50.0) 2 (10.0)	n.a.
**Pathological T Stage (%)** pT1 pT2 pT3 pT4 Not determined	30 (29.1) 50 (48.5) 3 (2.9) 5 (4.9) 15 (14.6)	5 (25.0) 13 (65.0) 0 (0.0) 1 (5.0) 1 (5.0)	n.a.
**Pathological N Stage (%)** pN0 p N1 p N2 p N3 Not determined	40 (38.8) 38 (36.9) 7 (6.8) 3 (2.9) 15 (14.6)	8 (40.0) 8 (40.0) 3 (15.0) 0 (0.0) 1 (5.0)	n.a.
**Adjuvant RT** Yes No Not determined	76 (73.8) 17 (16.5) 10 (9.7)	17 (85.0) 3 (15.0) 0 (0.0)	n.a.
**Adjuvant ChT** Yes No Not determined	37 (35.9) 50 (48.6) 16 (15.5)	11 (55.0) 7 (35.0) 2 (10.0)	n.a.

NBr, normal breast tissues; NST, no special type; IDC, invasive ductal carcinoma; HER2, human epidermal growth factor receptor 2; G, grade; RT, radiotherapy; ChT, chemotherapy; n.a., not applicable.

### Global Focus MiRNA PCR Panel Analysis

In the global expression assay, one luminal A case with recurrence was excluded from the analysis, due to low RT-qPCR success rate (25% of the miRNAs did not amplify, and the remaining showed Ct values higher than 30). Likewise, 3 (*miR-202-3p*, *-206*, and *-20b-5p*) out of the 80 miRNAs were excluded due to low real-time PCR success rates. MiRNAs with fold variation values higher than 1 were selected, resulting in a panel comprising 56 miRNAs (**Table 4**).

**Table 4:** MiRNAs with fold variation values higher than 1 in the global expression assay.

LumA Rec *vs*. LumA NRec		LumB Rec *vs*. LumB NRec		Lum Rec *vs*. Lum NRec	
microRNA	Fold Change	microRNA	Fold Change	microRNA	Fold Change
**miR-196a-5p**	2.1281	**miR-9-5p^1^**	2.5978	**miR-9-5p^1^**	1.4448
**miR-181b-5p**	−1.0119	**miR-210-3p^1^**	1.7178	**miR-149-3p^1^**	1.23995
**miR-130a-3p^1^**	−1.0519	**miR-182-5p^2^**	1.6028	**miR-126-3p**	−1.0909
**miR-29b-3p**	−1.1169	**miR-7-5p^1^**	1.3978	**miR-1**	−1.1352
**let-7b-5p**	−1.1269	**miR-200c-3p**	1.2778	**miR-148a-3p**	−1.1419
**let-7i-5p**	−1.1369	**miR-31-5p^1^**	1.0928	**miR-30d-5p**	−1.2139
**miR-106b-5p**	−1.1419	**miR-221-3p**	1.0128	**miR-181a-5p^2^**	−1.4322
**miR-132-3p^1^**	−1.1519	**miR-125b-5p**	−1.0172	**miR-200a-3p**	−1.5732
**miR-26b-5p**	−1.1619	**miR-146a-5p**	−1.0372	**miR-205-5p^2^**	−2.3252
**miR-19b-3p**	−1.1769	**miR-181a-5p^2^**	−1.0622		
**miR-192-5p^1^**	−1.1969	**miR-205-5p^2^**	−1.1172		
**let-7g-5p**	−1.2019	**miR-1^1^**	−1.1472		
**miR-16-5p**	−1.2319	**miR-10b-5p**	−1.4022		
**miR-15a-5p**	−1.2619				
**miR-106a-5p**	−1.2669				
**miR-20a-5p**	−1.2769				
**let-7a-5p**	−1.3019				
**miR-21-5p**	−1.3169				
**miR-214-3p**	−1.3569				
**miR-93-5p**	−1.4119				
**let-7f-5p**	−1.4369				
**miR-222-3p**	−1.4419				
**miR-200c-3p**	−1.4719				
**miR-155-5p**	−1.5119				
**let-7e-5p**	−1.5119				
**let-7d-5p**	−1.5619				
**miR-148a-3p**	−1.6369				
**miR-181a-5p^2^**	−1.6519				
**miR-23b-3p**	−1.7569				
**miR-23a-3p**	−1.8069				
**miR-19a-3p**	−1.8519				
**miR-1^1^**	−1.8869				
**miR-221-3p**	−1.9319				
**miR-195-5p**	−1.9369				
**miR-18a-5p^1^**	−1.9919				
**miR-30c-5p^2^**	−2.0419				
**miR-182-5p^2^**	−2.1119				
**miR-186-5p^1^**	−2.1319				
**miR-141-3p**	−2.1619				
**miR-17-5p^1^**	−2.1919				
**miR-30d-5p**	−2.2769				
**miR-30b-5p^2^**	−2.4819				
**miR-101-3p**	−2.5319				
**miR-200b-3p^2^**	−3.0019				
**miR-92b-3p^1^**	−3.1069				
**miR-200a-3p**	−3.2169				
**miR-205-5p^2^**	−4.1269				

^1^Cps higher than 30. ^2^miRNAs chosen for further validation. Lum, luminal; Rec, recurrent.

### Gene-Specific Assays

From the global expression assay analysis, *miR-30b-5p*, *miR-30c-5p*, *miR-181a-5p*, *miR-182-5p*, *miR-200b-3p*, and *miR-205-5p* were selected for further validation. All these miRNAs disclosed prominent fold change and good sensitivity for qRT-PCR detection, with different ranges of expression. *MiR-30b-5p* was chosen because several studies focused on other members of the *miR-30 family (miR-30f)* and, to the best of our knowledge, its predictive potential for ET had not been assessed previously ( Cheng et al., 2012; Bockhorn et al., 2013; Zhang et al., 2014b; D’aiuto et al., 2015; Yang et al., 2017). *MiR-181a-5p* and *miR-200b-3p* were selected to confirm the reported association with endocrine resistance in *in vitro* studies (Hiscox et al., 2006; Maillot et al., 2009; Manavalan et al., 2011; Vesuna et al., 2012; Manavalan et al., 2013). Furthermore, *miR-182-5p* was also selected to better ascertain its role in endocrine resistance due to controversial results in global focus miRNA PCR panel, since it was overexpressed in luminal B tumors from recurrent patients and downregulated in luminal A tumors from recurrent patients. Finally, *miR-30c-5p* was chosen as a positive control since higher expression levels of this miRNA had been positively associated with benefit of ET, in multivariate analysis, in advanced ER-positive BrC (Rodriguez-Gonzalez et al., 2011).

**Figure 1:**
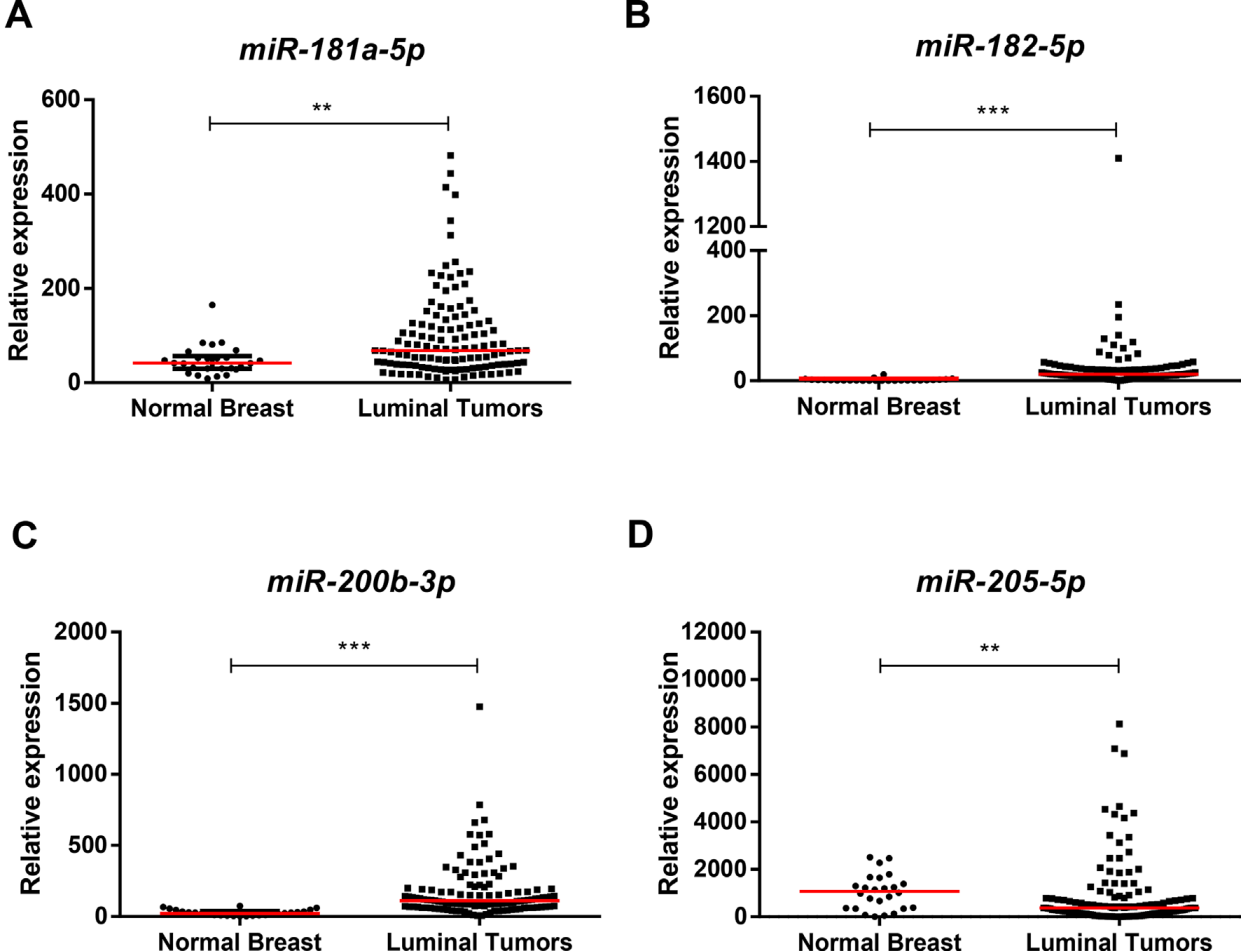
Scatterplots of *miR-181a-5p* **(A)**, *miR-182-5p* **(B)**, *miR-200b-3p* **(C)**, and *miR-205-5p* **(D)** relative expression levels in luminal tumor tissues and normal breast tissues. A ** denotes *p* value <0.01 and a *** denotes *p* value <0.001 by non-parametric Mann–Whitney *U* test. *Y*-axis denotes 2^−ΔΔCT^ values multiplied by 1000. Red horizontal lines represent median value.

**Figure 2:**
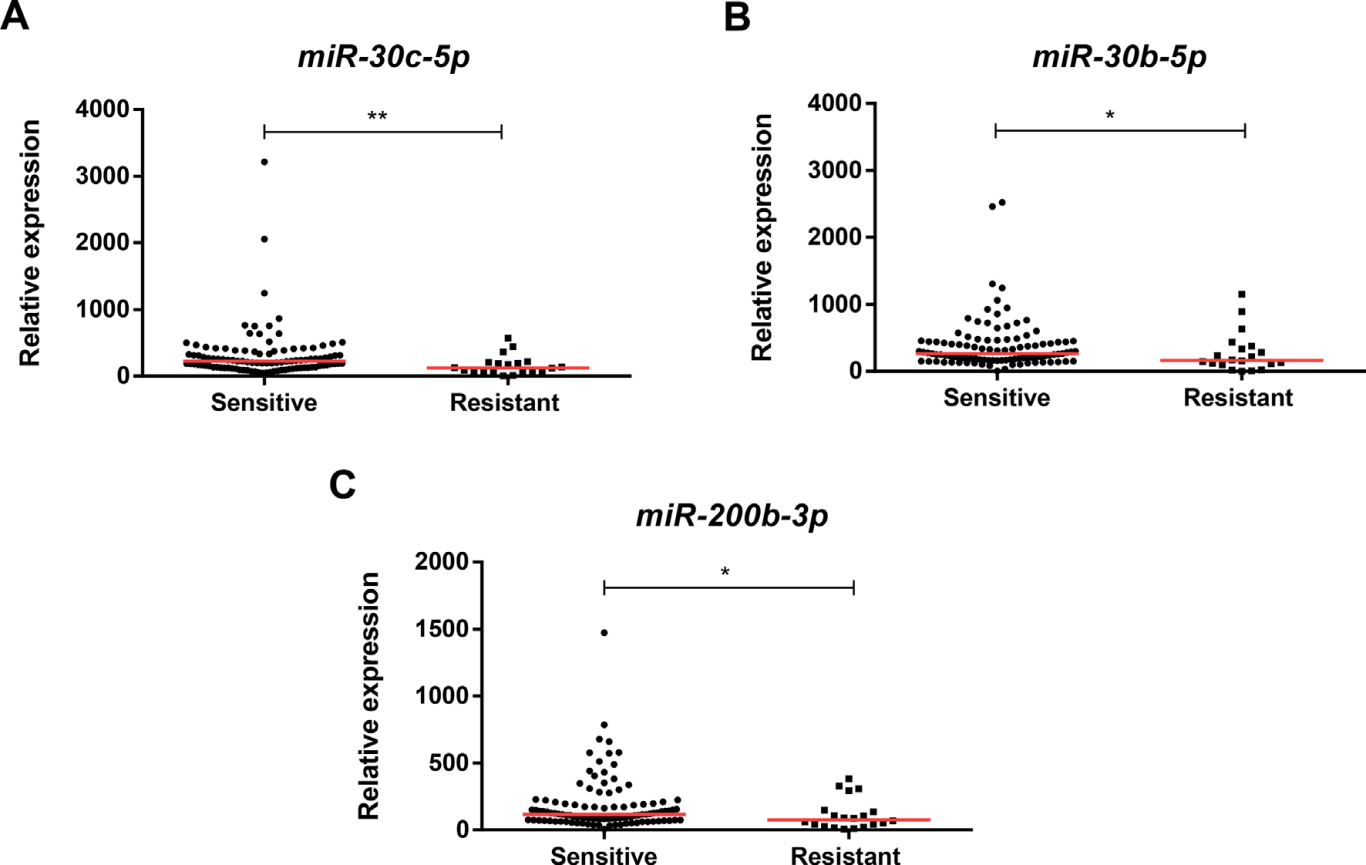
Scatterplots of *miR-30c-5p* **(A)**, *miR-30b-5p* **(B)**, and *miR-200b-3p* **(C)** relative expression levels in tumor tissues from endocrine-sensitive and -resistant patients. A * denotes *p* value <0.05 and a ** denotes *p* value <0.01 by non-parametric Mann–Whitney *U* test. *Y*-axis denotes 2^−ΔΔCT^ values multiplied by 1000. Red horizontal lines represent median value.

Except for *miR-205-5p* expression (*p* = 0.001), *miR-181a-5p* (*p* = 0.004), *miR-182-5p* (*p* < 0.001), and *miR-200b-3p* (*p* < 0.001), expression levels were significantly higher in luminal BrC tissues than in normal breast tissues (**Figure 1**), whereas no differences were depicted for the levels of the remaining miRNAs. Nonetheless, *miR-30b-5p* (*p* = 0.031), *miR-30c-5p* (*p* = 0.002), and *miR-200b-3p* (*p* = 0.021) were significantly downregulated in endocrine-resistant BrC samples compared to endocrine-sensitive tumors (**Figure 2**).

### Association Between MiRNA Expression and Clinicopathological Features

**Figure 3:**
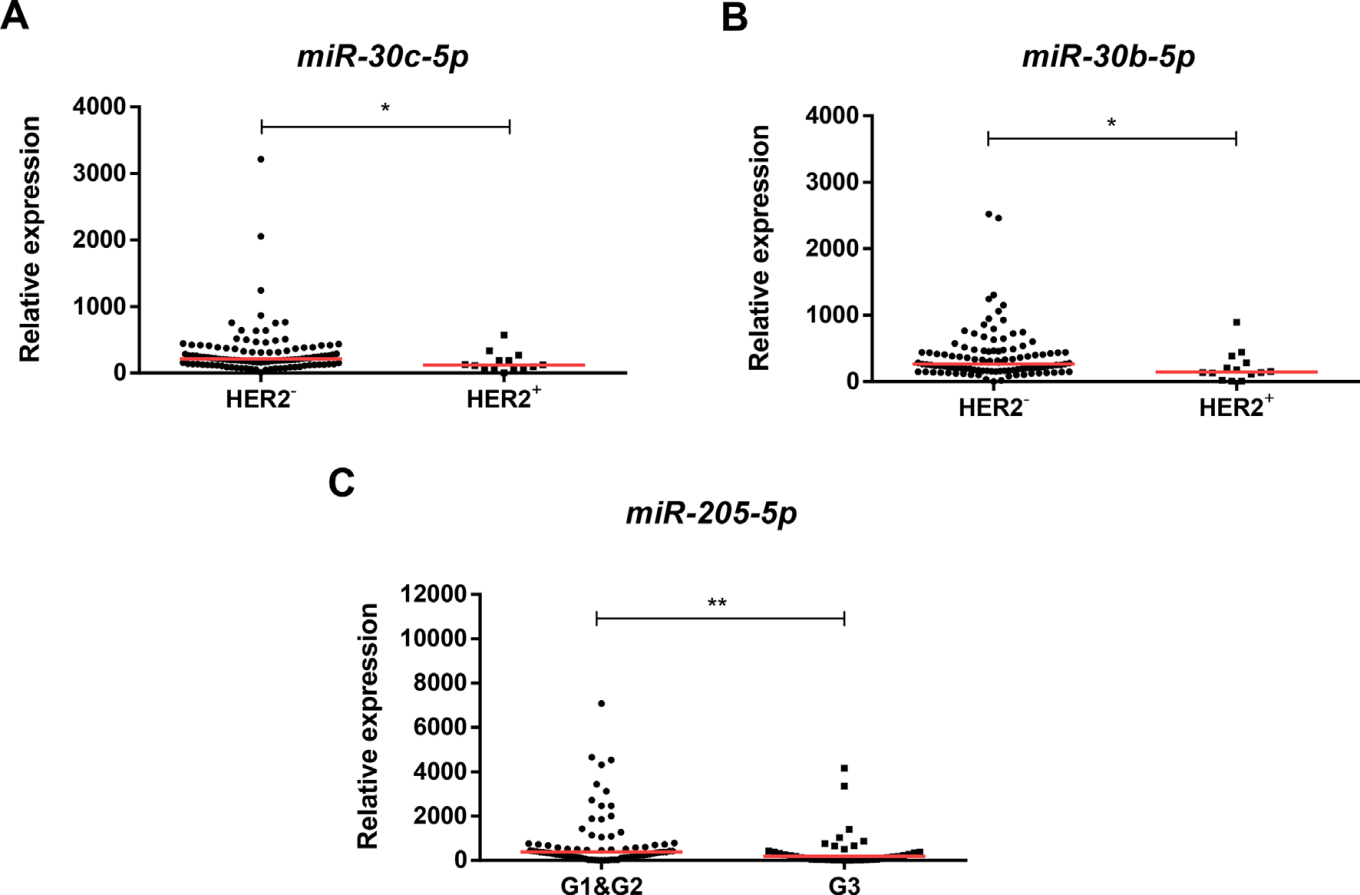
Scatterplots of *miR-30c-5p* relative expression levels according to HER2 *status* **(A)**, *miR-30b-5p* relative expression according to HER2 *status* **(B)**, and *miR-205-5p* relative expression according to grade **(C)**. A * denotes *p* value <0.05 and a ** denotes *p* value <0.01 by non-parametric Mann–Whitney *U* test. Y-axis denotes 2^−ΔΔCT^ values multiplied by 1000. Red horizontal lines represent median value.

Higher *miR-30b-5p* and m*iR-30c-5p* expression levels were found in tumors lacking HER2 overexpression (HER2-negative) (*p* = 0.010, *p* = 0.014, respectively). Conversely, lower *miR-205-5p* expression levels were found in high grade (G3) BrC (*p* = 0.009) compared to G1/G2 BrC (**Figure 3**). Moreover, *miR-205-5p* expression levels inversely correlated with patients’ age (*R* = −0.200, *p* = 0.027).

### Survival Analyses

The median follow-up time was 180 months (17.4–180 months). At 15 years of follow-up, 70 (56.9% of total) patients were alive, of whom 66 (53.7% of total) had no evidence of cancer. Moreover, from the 53 patients (43.1% of total) who died, death was due to BrC in 30 (24.4% of total).

**Figure 4:**
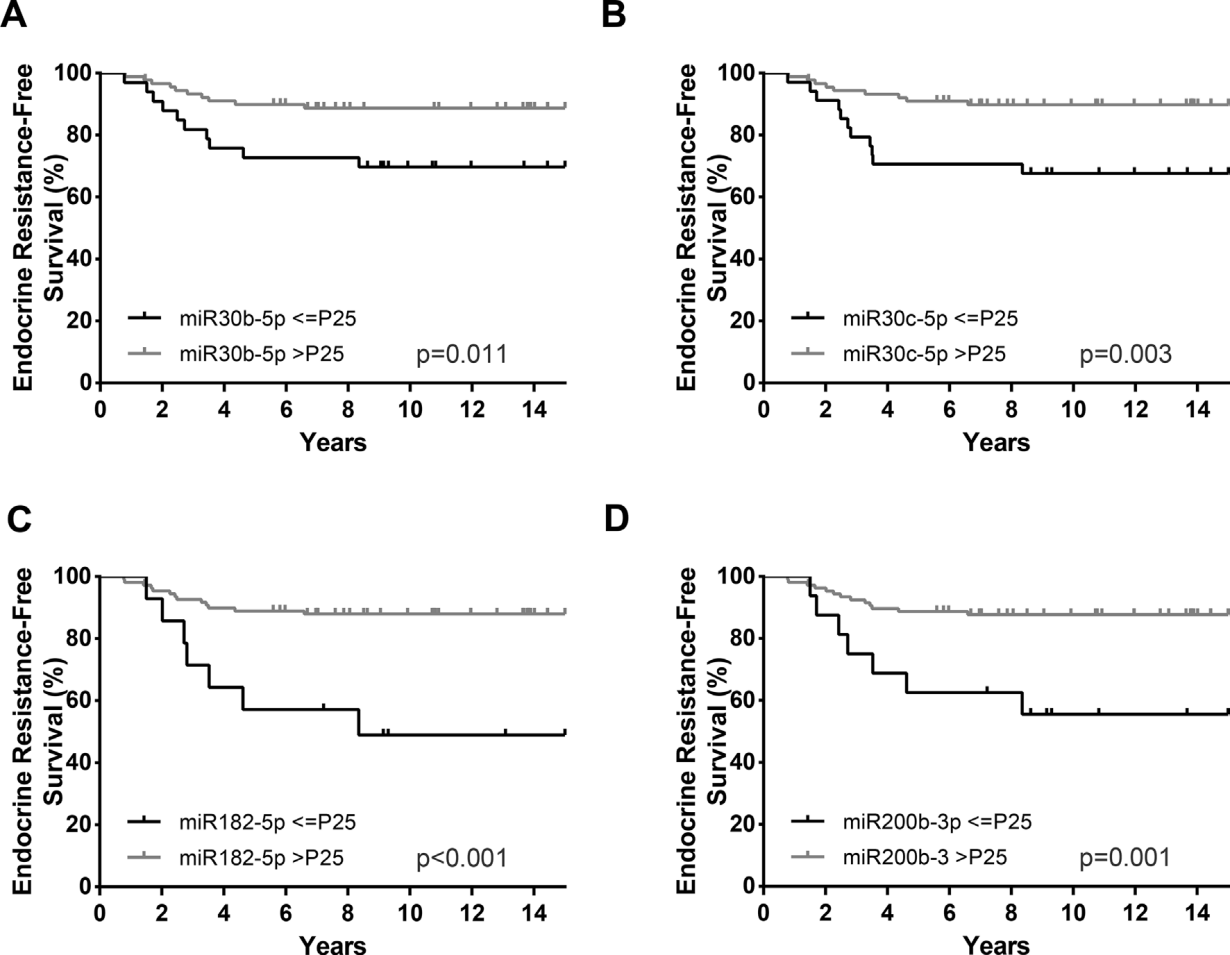
Endocrine resistance-free survival (ERFS) curves (Kaplan–Meier with log rank test) of *miR-30b-5p* **(A)**, *miR-30c-5p* **(B)**, *miR-182-5p* **(C)**, and *miR-200b-3p* **(D)**. P25, Percentile 25.

Overall, in univariable analysis, most standard clinicopathological parameters were significantly associated with ERFS. Specifically, patients with HER2 positivity (HR = 2.91, *p* = 0.039), high Ki-67 *index* (HR = 5.59, *p* = 0.001), high grade (G3) (HR = 2.84, *p* = 0.028), and luminal B subtype (HR = 4.48, *p* = 0.017) disclosed shorter ERFS. Importantly, the same was observed for patients with lower *miR-30c-5p*, *miR-30b-5p*, *miR-182-5p*, and *miR-200b-3p* levels (**Table 5**, **Figure 4**). In multivariable analysis, all miRNAs remained independent predictors of ERFS adjusted for Ki-67 *index* (**Table 5**). After stratification for Ki-67 *index*, *miR-30c-5p*, *miR-182-5p*, and *miR-200b-3p* only independently predicted shorter ERFS in highly proliferative tumors, whereas *miR-30b-5p* was significant in tumors with low proliferative (**Table 6**).

**Table 5:** Univariable and multivariable Cox regression models assessing the association between microRNAs expression levels and clinical outcome.

Model	Outcome	Variable	HR (95% CI)	*p* value
**Univariate Analysis**	**ERFS**	miR-30c-5p expression categorized ≤P25 >P25	1 0.283 (0.117–0.683)	0.005
		miR-30b-5p expression categorized ≤P25 >P25	1 0.338 (0.141–0.812)	0.015
		miR-182-5p expression categorized ≤P25 >P25	1 0.207 (0.082–0.519)	0.001
		miR-200b-3p expression categorized ≤P25 >P25	1 0.245 (0.098–0.615)	0.003
	**DFS**	miR-30c-5p expression categorized ≤P25 >P25	1 0.422 (0.214–0.832)	0.013
		miR-30b-5p expression categorized ≤P25 >P25	1 0.458 (0.231–0.907)	0.025
		miR-182-5p expression categorized ≤P25 >P25	1 0.259 (0.120–0.558)	0.001
		miR-200b-3p expression categorized ≤P25 >P25	1 0.267 (0.127–0.562)	0.001
		miR-205-5p expression categorized ≤P25 >P25	1 0.494 (0.250–0.979)	0.043
	**DMFS**	miR-182-5p expression categorized ≤P25 >P25	1 0.356 (0.153–0.828)	0.017
		miR-200b-3p expression categorized ≤P25 >P25	1 0.354 (0.158–0.794)	0.012
**Multivariate analysis**	**ERFS**	miR-30c-5p expression categorized^1^ ≤P25 >P25	1 0.223 (0.076–0.649)	0.006
		miR-30b-5p expression categorized^1^ ≤P25 >P25	1 0.344 (0.120–0.987)	0.047
		miR-182-5p expression categorized^1^ ≤P25 >P25	1 0.174 (0.057–0.529)	0.002
		miR-200b-3p expression categorized^1^ ≤P25 >P25	1 0.184 (0.060–0.561)	0.003
	**DFS**	miR-30c-5p expression categorized^1^ ≤P25 >P25	1 0.417 (0.193–0.902)	0.026
		miR-182-5p expression categorized^1^ ≤P25 >P25	1 0.190 (0.078–0.463)	<0.001
		miR-200b-3p expression categorized^1^ ≤P25 >P25	1 0.231 (0.094–0.564)	0.001
	**DMFS**	miR-182-5p expression categorized^2^ ≤P25 >P25	1 0.302 (0.111–0.825)	0.020

^1^Cox regression model adjusted for Ki-67 index. ^2^Cox regression models adjusted for grade. ERFS, endocrine resistance-free survival; DFS, disease-free survival; DMFS, distant metastasis-free survival.

**Table 6:** Cox regression models stratified according to the clinicopathological features with statistical significance in the multivariate analysis.

Outcome	Layering Variable	Variable	HR (95% CI)	*p* value
**ERFS**	Ki-67 index <15%	miR-30c-5p expression categorized ≤P25 >P25	–	0.175
	Ki-67 index >15%	miR-30c-5p expression categorized ≤P25 >P25	1 0.171 (0.047–0.619)	0.007
	Ki-67 index <15%	miR-30b-5p expression categorized ≤P25 >P25	1 0.149 (0.027–0.813)	0.028
	Ki-67 index >15%	miR-30b-5p expression categorized ≤P25 >P25	–	0.334
	Ki-67 index <15%	miR-182-5p expression categorized ≤P25 >P25	–	0.537
	Ki-67 index >15%	miR-182-5p expression categorized ≤P25 >P25	1 0.137 (0.037–0.503)	0.003
	Ki-67 index <15%	miR-200b-3p expression categorized ≤P25 >P25	–	0.610
	Ki-67 index >15%	miR-200b-3p expression categorized ≤P25 >P25	1 0.121 (0.033–0.447)	0.002
**DFS**	Ki-67 index <15%	miR-30c-5p expression categorized ≤P25 >P25	–	0.247
	Ki-67 index >15%	miR-30c-5p expression categorized ≤P25 >P25	1 0.268 (0.088–0.815)	0.020
	Ki-67 index <15%	miR-182-5p expression categorized ≤P25 >P25	–	0.141
	Ki-67 index >15%	miR-182-5p expression categorized ≤P25 >P25	1 0.137 (0.037–0.503)	0.003
	Ki-67 index <15%	miR-200b-3p expression categorized ≤P25 >P25	–	0.202
	Ki-67 index >15%	miR-200b-3p expression categorized ≤P25 >P25	1 0.121 (0.033–0.447)	0.002
**DMFS**	Grades 1 and 2	miR-182-5p expression categorized ≤P25 >P25	1 0.255 (0.079–0.823)	0.022
	Grade 3	miR-182-5p expression categorized ≤P25 >P25	–	0.076

ERFS, endocrine resistance-free survival; DFS, disease-free survival; DMFS, distant metastasis-free survival; HER2, human epidermal growth factor 2 receptor.

**Figure 5:**
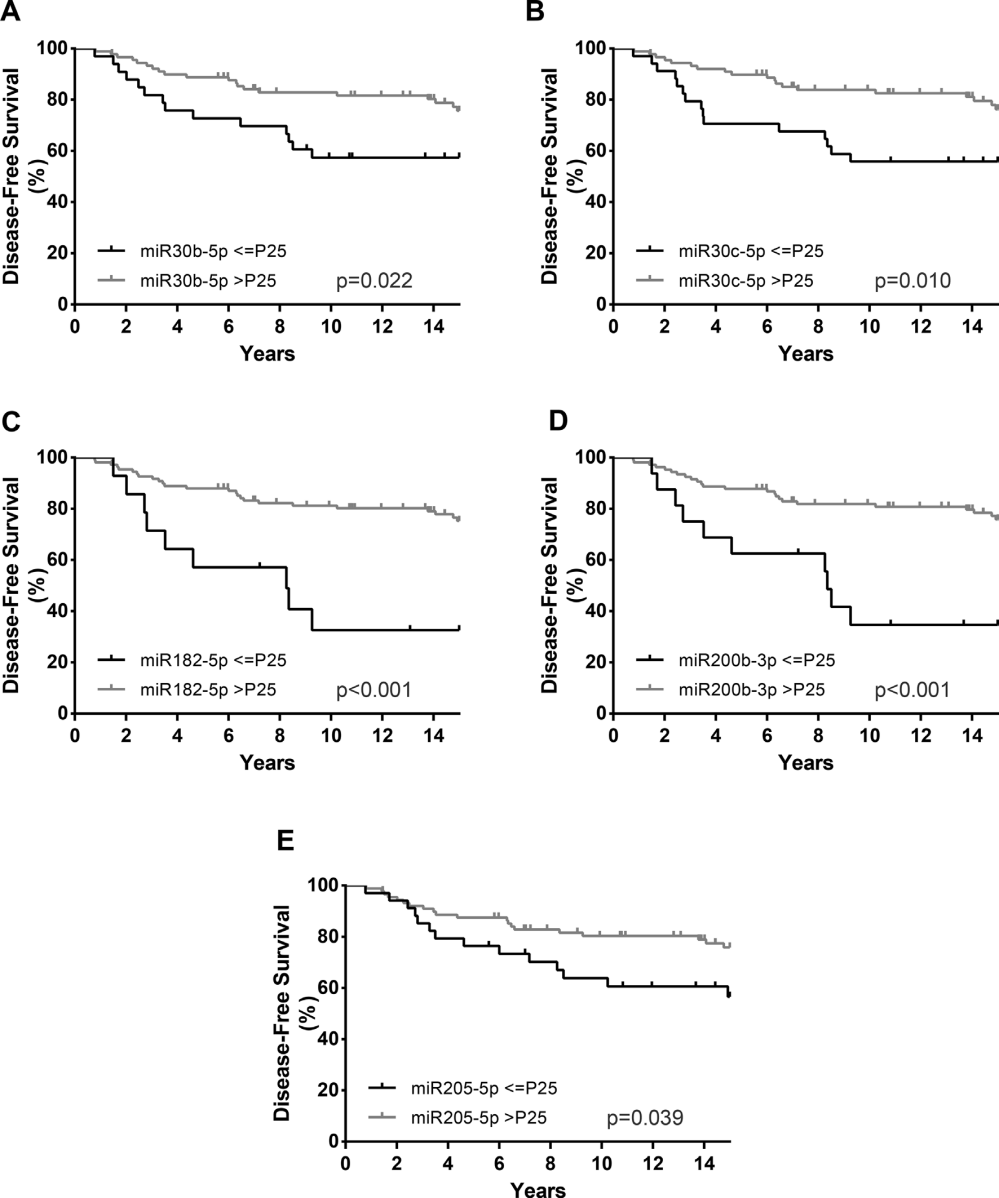
Disease-free survival (DFS) curves (Kaplan–Meier with log rank test) of *miR-30b-5p* **(A)**, *miR-30c-5p* **(B)**, *miR-182-5p* **(C)**, *miR-200b-3p* **(D)**, and *miR-205-5p* **(E)**. P25, Percentile 25.

Regarding DFS, in addition to HER2 positivity (HR = 2.40, *p* = 0.039), high Ki-67 *index* (HR = 3.01, *p* = 0.003), and high grade (G3) (HR = 2.65, *p* = 0.006), lower *miR-30c-5p*, *miR-30b-5p*, *miR-182-5p*, *miR-200b-3p*, and *miR-205-5p* expression levels associated with decreased DFS in univariable analysis (**Table 5**, **Figure 5**). Nonetheless, in the multivariable model, only *miR-30c-5p*, *miR-200b-3p*, and *miR-182-5p* were disclosed as independent prognostic predictors adjusted for Ki-67 *index* (**Table 5**), and after stratification according for Ki-67 *index*, all miRNAs retained statistical significance in high Ki-67 *index* BrC patients (**Table 6**). Similarly, HER2 positivity (HR = 2.63, *p* = 0.024), high Ki-67 *index* (HR = 2.48, *p* = 0.021), and high grade (G3) (HR = 2.69, *p* = 0.007) associated with worse DMFS, along with lower *miR-182-5p* and *miR-200b-3p* expression levels, in univariate analysis (**Table 5**). However, only *miR-182-5p* retained statistical significance when adjusted for tumor grade in multivariable analysis (**Table 5**). After stratification by tumor grade, *miR-182-5p* showed prognostic value in patients harboring low/intermediate-grade tumors (**Table 6**).

**Figure 6:**
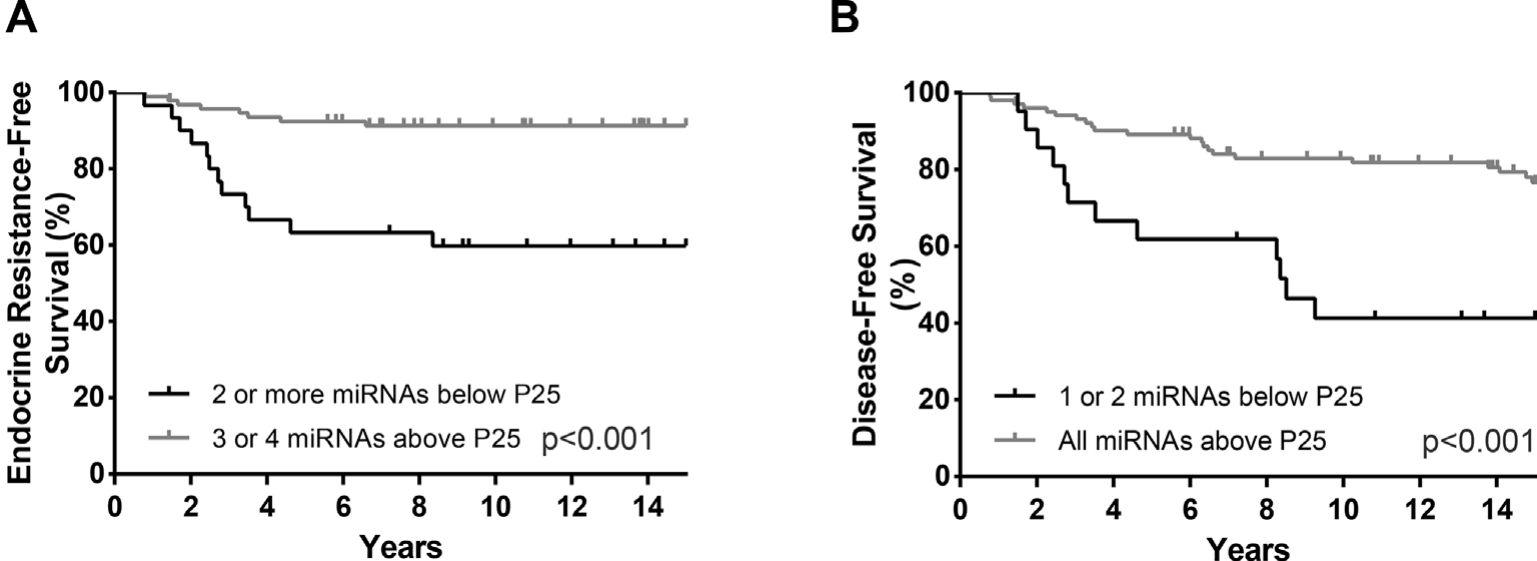
ERFS curves (Kaplan–Meier with log rank test) of combined *miR-30b-5p*, *miR-30c-5p*, *miR-182-5p* and *miR200b-3p* **(A)** and DFS curves (Kaplan–Meier with log rank test) of combined *miR-182-5p* and *miR-200b-3p* panel **(B)**.

Furthermore, the prognostic value of the miRNAs that individually predicted ERFS and DFS was assessed when combined in panels. For ERFS, the patients were grouped as expression above P25 for 3 or 4 miRNAs versus expression below P25 for 2 or more miRNAs. Thus, the combination of m*iR-30c-5p*, *miR-30b-5p*, *miR-182-5p*, and *miR-200b-3p* was shown as the best predictors of ERFS. Patients with miRNAs’ expression below P25 displayed a shorter ERFS (*p* < 0.001), paralleling the results obtained in single miRNAs analysis (**Figure 6**, **Table 7**). In multivariable analysis, miRNAs combined in panel were found to be independent ERFS predictors after Ki-67 *index* stratification (**Table 7**). Regarding DFS, the best predictive panel was composed of *miR-182-5p* and *miR-200b-3p*. The patients were grouped as expression above P25 for both miRNAs versus expression below P25 for at least one miRNA. Patients with both miRNAs’ expression levels above P25 showed longer DFS (*p* < 0.001) (**Figure 6**, **Table 7**). In multivariable analysis, miRNAs combined in panel remained independent DFS predictors, although only in cases with high Ki-67 *index* (**Table 7**).

**Table 7:** Univariable and multivariable Cox regression models assessing the association between combined microRNAs expression panel and clinical outcome.

Model	Outcome	Layering Variable	Variable	HR (95% CI)	*p* value
**Univariate Analysis**	ERFS	NA	**Combined miRNA panel** 2 or more miRNAs below P25 3 or 4 miRNAs above P25	1 0.183 (0.075–0.448)	<0.001
	DFS	NA	**Combined miRNA panel** 1 or 2 miRNAs below P25 All miRNAs above P25	1 0.283 (0.139–0.575)	<0.001
**Multivariate Analysis**	ERFS	NA	**Combined miRNA panel** ^1^ 2 or more miRNAs below P25 3 or 4 miRNAs above P25	1 0.126 (0.042–0.380)	<0.001
	DFS	NA	**Combined miRNA panel** ^1^ 1 or 2 miRNAs below P25 All miRNAs above P25	1 0.205 (0.088–0.476)	<0.001
**Multivariate Analysis**	ERFS	Ki-67 *index* <15%	**Combined miRNA panel** 2 or more miRNAs below P25 3 or 4 miRNAs above P25	1 0.129 (0.024–0.703)	0.018
		Ki-67 *index* >15%	**Combined miRNA panel** 2 or more miRNAs below P25 3 or 4 miRNAs above P25	1 0.134 (0.037–0.485)	0.002
	DFS	Ki-67 *index* <15%	**Combined miRNA panel** 1 or 2 miRNAs below P25 All miRNAs above P25	–	0.184
		Ki-67 *index* >15%	**Combined miRNA panel** 1 or 2 miRNAs below P25 All miRNAs above P25	1 0.100 (0.027–0.367)	0.001

^1^Cox regression model adjusted for Ki-67 index. ERFS, endocrine resistance-free survival; DFS, disease-free survival; NA, not applicable.

## Discussion

BrC remains the most common malignancy in women and a major cause of morbidity and mortality (Bray et al., 2018). De-escalation of both systemic and local adjuvant treatment, paralleling trends in surgery, is critical to provide patient-tailored treatment and avoid harmful side effects (Hwang, 2014; Senkus et al., 2015). Indeed, identification of luminal BrC patients with low recurrence risk after or while on ET, for which additional adjuvant systemic treatment can be safely omitted, is very important. On the other hand, identification of high-risk luminal BrC patients requiring more aggressive treatment regimens is critical to avoid recurrence and subsequent metastatic disease, which currently affects approximately 40% of luminal BrC patients after adjuvant ET (Guarneri and Conte, 2004; Normanno et al., 2005; Murphy and Dickler, 2016). Thus, identification of biomarkers providing predictive and prognostic information in this group of patients is clinically relevant. Assessment of specific miRNAs’ expression deregulation, which has been associated with several mechanisms underlying endocrine resistance and sensitivity (Muluhngwi and Klinge, 2015; Muluhngwi and Klinge, 2017), might provide such kind of information. Nonetheless, most of those studies have been performed in cancer cell lines and display several limitations, including absence of epithelial–stromal and tumor–host interactions, that could modulate sensitivity *in vivo* (Shekhar et al., 2003). Conversely, tissue analysis from patients treated with ET may allow for broader insight into biologically and clinically relevant miRNAs that may serve as markers of response or resistance to ET. Thus, we focused on the identification of aberrantly expressed miRNAs in endocrine-resistant BrC, exploring its predictive and prognostic value in luminal BrC patients treated with adjuvant ET.

The first step of this study consisted on the profiling of miRNAs’ expression patterns, looking for differences between endocrine-sensitive and endocrine-resistant luminal BrC. Hence, *miR-30c-5p*, *miR-30b-5p*, *miR-181a-5p*, *miR-182-5p*, *miR-200b-3p*, and *miR-205-5p* were selected for validation in a larger set of luminal BrC and normal breast tissues. Upregulation of *miR-181a-5p* and *miR-182-5p* and downregulation of *miR-205-5p* in this BrC tissue cohort was consistent with previous reports (Hui et al., 2009; Li et al., 2014a; Zhang and Fan, 2015), providing indirect validation of our methodological approach. However, *miR-200b-3p* downregulation in tumor compared to normal tissues has been previously reported (Ye et al., 2014; Yao et al., 2015). Nevertheless, these studies have used non-cancerous tissues from breasts harboring carcinoma as controls, which may not represent truly normal breast tissues. Our results also confirm the biomarker potential of *miR-30c-5p*, which was found downregulated in endocrine-resistant BrC patients and independently predicted ERFS in luminal BrC patients, particularly in highly proliferative tumors. Moreover, *miR-30c-5p* expression correlated with HER2 *status*, one of the most important predictive factors for ET sensitivity (Konecny et al., 2003). In fact, HER2 signaling activation has been widely implicated in endocrine resistance (Moon et al., 2011; AlFakeeh and Brezden-Masley, 2018). Moreover, *miR-200b-3p* expression levels displayed the same trend and, together with *miR-30b-5p* and *miR-182-5p*, also independently predicted ERFS in luminal BrC patients. Importantly, we were able to validate in primary BrC the association between *miR-200b-3p* and endocrine-resistance, which was previously reported only in *in vitro* models (Manavalan et al., 2013). Interestingly, several members of *miR-30f* have been reported as markers of favorable prognosis in BrC (Cheng et al., 2012; Bockhorn et al., 2013; Zhang et al., 2014b; D’aiuto et al., 2015; Croset et al., 2018) and our study also revealed that *miR-30b-5p* might be predictive of response to ET. Finally, concerning *miR-182-5p*, our results extended previous observations on the correlation with clinical benefit from therapy with tamoxifen in advanced-stage BrC, only previously demonstrated in univariable analysis (Rodriguez-Gonzalez et al., 2011).

In addition to their predictive value, *miR-30b-5p* and *miR-30c-5p* lower expression levels also associated with decreased DFS, although in univariable analysis only. Indeed, the role of m*iR-30f* members as tumor suppressors in BrC has been previously reported (Bockhorn et al., 2013; Zhang et al., 2014b). Furthermore, decreased levels of *miR-30f* members in BrC patients have been associated with poor relapse-free survival (Croset et al., 2018). Importantly, lower *miR-182-5p* and *miR-200b-3p* expression levels independently associated with decreased DFS in highly proliferative tumors. The role of *miR-200b-3p* as a prognostic marker in BrC is not a novelty (Ye et al., 2014; Yao et al., 2015). Indeed, members of *miR-200f* are known to act as enforcers of epithelial phenotype through either Zinc finger E-box-binding homeobox (ZEB)-dependent or -independent pathways (Li et al., 2014b). Intriguingly, most *in vitro* studies consistently attributed an oncogenic role to *miR-182-5p* (Chiang et al., 2013; Zhan et al., 2017). Nonetheless, higher *miR-182-5p* expression levels were associated with poor clinical outcome in BrC patients (Song et al., 2016), contrarily to our findings. It should be recalled, however, that *miR-182-5p* is a member of a miRNA family comprising three homologous, coordinately expressed, miRNAs (*miR-183*, *miR-182*, and *miR-196*), which are clustered in chromosome 7q32.2 and that members of this cluster have been associated with both pro- and anti-metastatic behavior in BrC, suggesting that *miR-183/96/182* cluster members may have divergent functions that are regulated in a context- and tissue-dependent manner (Lowery et al., 2010; Li et al., 2014a; Hong et al., 2016). Furthermore, the 7q32.2 locus has been considered a metastasis suppressor locus, enduring genetic copy number losses in BrC progression (Png et al., 2011). Thus, the association between *miR-182-5p* downregulation and worse prognosis probably results from a complex molecular scenario and additional studies are required to discriminate which members of the *miR-183/96/182* cluster might contribute and to which extent to BrC prognosis.

BrC tissues displayed higher *miR-182-5p* and *miR-200b-3p* levels compared to normal breast, although *miR-182-5p* and *miR-200b-3p* downregulation associated with shorter DMFS. Because development of solid neoplasms results from multiple sequential steps in which malignant cells undergo widespread modifications allowing for successful migration and colonization of other organs, we are tempted to speculate whether a context-dependent role of these miRNAs might contribute to the emergence of a malignant phenotype. Indeed, decreased expression of *miR-200f* members might be associated with EMT initiation, enabling cells with invasive capabilities, whereas subsequent upregulation might be associated with mesenchymal-to-epithelial transition, facilitating colonization (Gravgaard et al., 2012; Hilmarsdottir et al., 2014).

Combined expression levels of *miR-30c-5p*, *miR-30b-5p*, *miR-182-5p*, and *miR-200b-3p* independently predicted ERFS, when adjusted for confounding factors (Ki-67 *index*). In fact, this combined miRNA panel was associated with ERFS in both low and highly proliferative tumors. In parallel, the *miR-182-5p*/*miR-200b-3p* panel was shown to independently predict DFS in highly proliferative tumors. As previously reported in different tumor models, the combination of miRNAs in a panel might enable a more efficient diagnostic, predictive, and prognostic model overcoming the questionable value of single miRNAs (Sahlberg et al., 2015; Chen et al., 2018).

Although the retrospective design of the study and the relatively small number of samples of the discovery cohort constitute important limitations, our results suggest that a panel of miRNAs might be tested in primary tumor tissues to assess the likelihood of recurrence and resistance to ET in newly diagnosed luminal BrC. Nevertheless, these miRNAs need to be carefully validated, ideally in multicenter studies, to generate more conclusive results. Furthermore, *in vitro* studies, including gain- and loss-of-function assays following *in vitro* treatment with ET, are also critical to functionally characterize the role of these miRNAs. As a future perspective, we intend to evaluate the putative role of these miRNAs in tumor progression and dissemination. Additionally, we also intend to evaluate the potential role of these miRNAs in liquid biopsies, evaluating their potential as non-invasive biomarkers. Indeed, miRNAs in circulation would enable the repeated noninvasive monitoring of miRNA expression profile changes during treatment’s course, which could allow for early detection of ET resistance and/or recurrence, potentially improving the management and care of luminal BrC patients.

## Ethics Statement

This study was carried out in accordance with the recommendations of Comissão de ética para a Saúde of Portuguese Oncology Institute of Porto, Portugal (CES-IPOFG-120/015) with written informed consent from all subjects. All subjects gave written informed consent in accordance with the Declaration of Helsinki. The protocol was approved by the Comissão de ética para a Saúde of Portuguese Oncology Institute of Porto, Portugal.

## Author Contributions

MA prepared tissues for molecular analyses, including RNA extraction and cDNA synthesis, performed RT-qPCR assays, analyzed data, and drafted the manuscript. JL and NC collected normal breast tissues from reductive mammoplasty and assisted in histopathological evaluation of tissue samples. HE-P and SS contributed in data analysis and in the manuscript preparation. MF-S and SPS collected clinical follow-up data. PL performed IHC of all cases. LA assisted in the statistical analyses. RH performed histopathological evaluation of fresh frozen sections stained by H&E. RH and CJ designed and supervised the study and revised the manuscript. All the authors read and approved the final manuscript.

## Funding

This work was supported by a grant from the Research Center of Portuguese Oncology Institute—Porto (PI 74-CI-IPOP-19-2016) and the Portuguese Society of Oncology—YOuR Project. SS is supported by a PhD fellowship IPO/ESTIMA-1 NORTE-01-0145-FEDER-000027. JL is supported by a PhD fellowship from FCT—Fundação para a Ciência e Tecnologia (SFRH/BD/132751/2017).

## Conflict of Interest Statement

The authors declare that the research was conducted in the absence of any commercial or financial relationships that could be construed as a potential conflict of interest.
