# Supplementary material for: Predictive and Prognostic Value of Selected MicroRNAs in Luminal Breast Cancer
Source: Front Genet. 2019 Sep 11;10:815. doi: 10.3389/fgene.2019.00815 (PMC6749838; doi:10.3389/fgene.2019.00815)
Supplement: Supplementary file 1 [file DataSheet_1.docx]

Predictive and prognostic value of selected microRNAs in luminal breast cancer

Amorim et al, Supplementary Material

# Supplementary Tables

Supplementary Table 1 - Specific target sequence of mature miRNAs tested.

| **Gene** | **Target sequence** | **MirBase accession** |
| --- | --- | --- |
| **SNORD38B (hsa)** | UCUCAGUGAUGAAAACUUUGUCCAGUUCUGCUACUGACAGUAAGUGAAGAUAAAGUGUGUCUGAGGAGA | NA |
| **hsa-miR-30b-5p** | UGUAAACAUCCUACACUCAGCU | MIMAT0000420 |
| **hsa-miR-30c-5p** | UGUAAACAUCCUACACUCUCAGC | MIMAT0000244 |
| **hsa-miR-181a-5p** | AACAUUCAACGCUGUCGGUGAGU | MIMAT0000256 |
| **hsa-miR-182-5p** | UUUGGCAAUGGUAGAACUCACACU | MIMAT0000259 |
| **hsa-miR-200b-3p** | UAAUACUGCCUGGUAAUGAUGA | MIMAT0000318 |
| **hsa-miR-205-5p** | UCCUUCAUUCCACCGGAGUCUG | MIMAT0000266 |
| NA-Not applicable | | |

Supplementary Figures

**
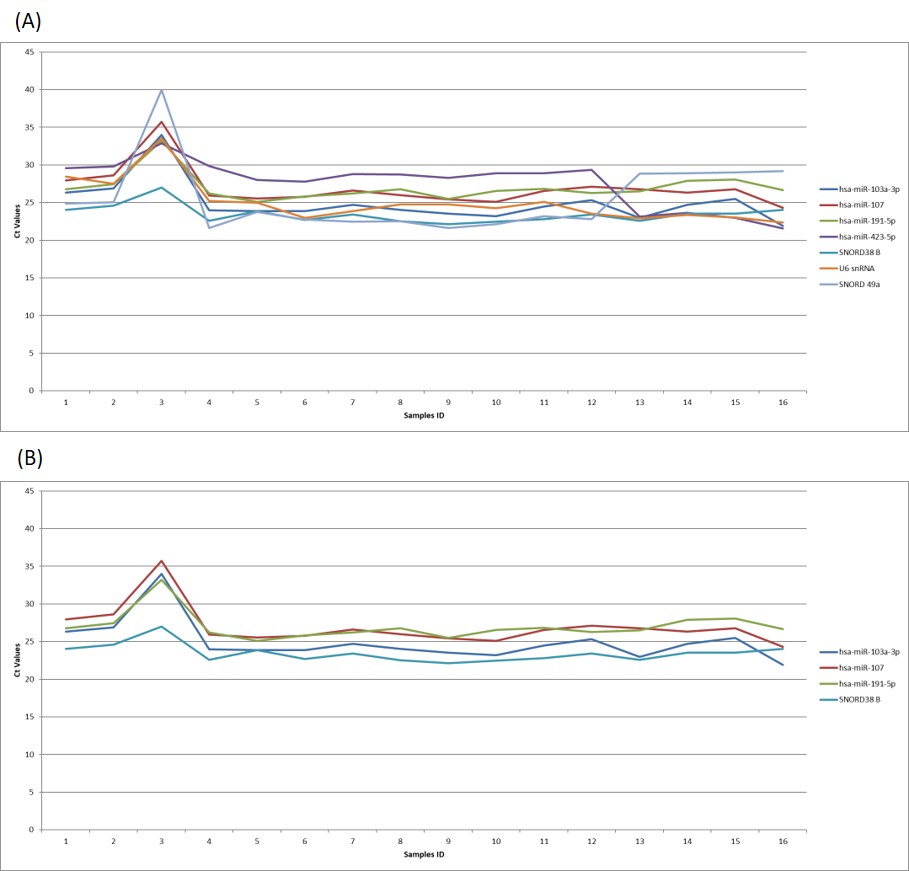
**

**Supplementary Figure 1.** **(A)** Ct values across the samples for the candidate reference genes. **(B)** Ct values across the samples for the selected reference genes.
